# Clearing the Smoke Screen: Smoking, Alcohol Consumption, and Stress Management Techniques among Canadian Long-Term Care Workers

**DOI:** 10.3390/ijerph17176027

**Published:** 2020-08-19

**Authors:** Iffath Unissa Syed

**Affiliations:** Faculty of Health, York University, 4700 Keele Street, Toronto, ON M3J 1P3, Canada; iusyed@yorku.ca

**Keywords:** public health, health behaviors, health promotion, long-term care, nursing homes, mixed-methods design, social determinants of health, gender

## Abstract

Background: Currently, there is abundant research indicating that smoking and alcohol consumption have significant impacts on morbidity and mortality, though little is known about these behaviors among Canadian health care workers. The objective of this study was to examine health and coping behaviors, such as smoking and alcohol consumption as well as stress management techniques, among health care workers consisting of gendered, racialized, and immigrant employees. Methods: Drawing on a single-case, mixed-methods study in Ontario, Canada, this paper presents under-researched data about smoking practices, alcohol consumption, and stress management techniques among health care workers in labor-intensive, high-stress, high-turnover environments. In particular, it identifies the various mechanisms for maintaining health and well-being. Results: The findings suggest that 7.7% of survey respondents reported smoking while 43.4% reported alcohol consumption, which were reported more frequently among immigrants than among non-immigrants. Participants also reported health-promoting activities in face-to-face interviews, such as mindful breathing techniques and drawing upon social support, while a few respondents reported alcohol consumption to specifically cope with sleep disturbances and job stress. Conclusions: Although smoking and alcohol consumption were both connected with coping strategies and leisure, they were predominant in immigrant groups compared to non-immigrant groups.

## 1. Introduction

Smoking and alcohol consumption can have problematic consequences on human health and they also impose economic costs to society. Smoking is responsible for more deaths than obesity, physical inactivity, or hypertension [1], resulting in 603,000 premature deaths worldwide [2], with work exposures claiming over 200,000 lives [3,4]. Smoking is associated with an increased risk of morbidity and mortality due to chronic obstructive pulmonary disease, asthma, cardiovascular disease, including ischemic, rheumatic, hypertensive, and inflammatory heart disease, as well as tracheal, bronchial, and lung cancers [1,5,6,7,8,9]. In Canada, it is estimated that smoking is attributed to 17% of all deaths and claims over 36,500 lives each year [5]. In addition to this, smoking-related illness kills 100 Canadians each day [5]. The economic cost of smoking was $16.2 billion in 2012 in Canada alone, with the largest component consisting of health care costs at roughly $6.5 billion [10]. These costs included prescription drugs ($1.7 billion), physician care ($1.0 billion), and hospital care ($3.8 billion) [10]. The federal, provincial, and territorial governments also spent $122.0 million on tobacco control and law enforcement [10].

As with the case of smoking, indicated above, alcohol consumption can also pose problems. Alcohol consumption is associated with esophageal cancer, liver cirrhosis, liver cancer, as well as toxicity, and accidental deaths, among other things [11,12]. It is estimated that alcohol use causes 2.5 million deaths each year around the world [13] and leads to 370,000 deaths due to road injuries, 150,000 deaths due to self-harm, and 90,000 deaths due to interpersonal violence [14]. “Mortality resulting from alcohol consumption is higher than that caused by diseases such as tuberculosis, HIV/AIDS and diabetes” [13]. Between 4 and 5 million Canadians engage in high-risk alcohol consumption [15], which is responsible for 40% of motor vehicle accidents in Canada [16].

Previous research suggests that certain health behaviors, coping strategies, and defense mechanisms are often adaptation processes in response to stress, and include escaping, avoidance, disengaging behaviors, and diminished health-promoting behaviors such as reduced adherence to health-promotion practices, dietary restrictions, and smoking cessation [17]. Indeed, occupational stress may have a significant effect on smoking and alcohol consumption. For example, occupational stress may result in the use of alcohol and cigarettes as anti-anxiety and anti-depressive agents to release job stress [18] and result in excessive smoking, increased smoking intensity, and increased alcohol consumption, which can be modulated by coping abilities [19]. Research on Canadian immigrants shows that these groups do not report elevated smoking intensity and alcohol consumption as coping strategies in response to work-related stress [19].

While it has been well known that both smoking and alcohol consumption can be utilized as coping mechanisms to stress responses [20,21], little is known about these consumption patterns and various other coping behaviors among workers who are employed in high-stress, high-turnover environments. In addition, little is known about these activities in long-term care (LTC) workers, who often comprise a gendered, racialized, and immigrant workforce. The rationale for selecting the study population consisting of health care workers was because previous research suggests that they face significant stress on a daily basis [22,23]. Health care workers’ coping and defense mechanisms are even more important now due to the current circumstances surrounding the COVID-19 pandemic that may result in increased stress from health workers’ own health risks to COVID-19, as well as the potential for additional stress from dealing with greater morbidity and mortality related to COVID-19 on the frontlines of care. Thus, studying the coping and defense mechanisms of health care workers is extremely important. Accordingly, the objective of this study was to examine routine health and coping behaviors, such as smoking and alcohol consumption as well as stress management techniques among health care workers. The rationale for selecting this specific group of workers was to gather knowledge about this population in order to fill knowledge gaps.

## 2. Materials and Methods

**Figure 1:**
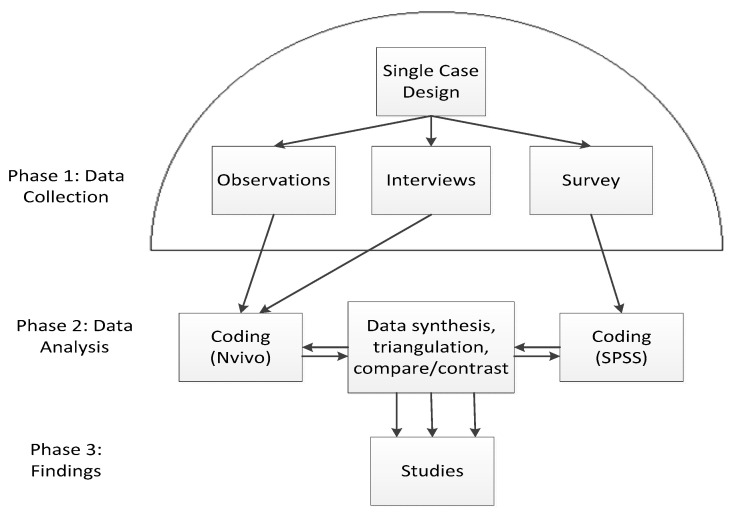
Flow diagram.

This study was derived from a larger project that utilized a single-case research design, which involved qualitative interviews, observations, and a survey for quantified description of LTC workers (Figure 1). The site was selected based on the feasibility of the project, richness of data, and geographical boundaries in an urbanized region that is known as a settlement destination for immigrants and visible minorities (VMs). A single-case study design was selected in order to examine the following, among other things: how do VMs and/or immigrant employees experience work compared to non-VM, non-immigrant employees in residential LTC? In what ways (and why) are these experiences distinct? Are there gender differences? These broad questions were developed from the use of feminist lenses and critical race theory, which guided the inquiry. Feminists argue that society is gendered in such a way that women and men have fundamentally different experiences and access to power and privilege [24]. Critical race theorists often focus on racialization, which is an active and ongoing process in which a dominant group identifies another group as having a race, often based on the latter group’s physical characteristics, such as skin color [25]. Racialization frequently results in unequal and unfair treatment of particular groups of women and men [26], and it has social, economic, and political consequences [27].

The site had a total of 176 workers. Sampling and recruitment for this study was broad and included non-VMs and men in order to compare and contrast experiences. The recruitment methods were snowball techniques and direct purposeful recruitment by the principal investigator. Ethics approval was received from York University’s Office of Research Ethics (ORE) (STU2016-139), and participant consent was obtained in writing and signed.

An ethnography was the primary data collection technique in which the sources of evidence were from direct observations and in-depth, key informant interviews. Observations and interviews were carried out by the researcher over two and a half months at a single LTC site between the hours of 6:30 a.m. and midnight, which were opportunistic times to observe the workers’ scheduled shift changes that occurred at 7 a.m., 3 p.m., and 11 p.m. Observations were conducted in secure, locked and unlocked units/wings at the site; in public spaces within the facility; and at the reception area. These spaces included hallways and dining areas on the individual units, a recreation space of the atrium located on the ground floor, an employee break room located at the mezzanine level, and spaces outside meeting rooms that were located in the basement level of the building. Fieldnotes were generated during observations, which documented preliminary thoughts, assumptions, and the physical setting. Observations sometimes included workers who had already participated in interviews. This was a way to trace and track the status of workers, e.g., immigrant/non-immigrant.

Face-to-face, in-depth, semi-structured interviews were conducted with participants and digitally recorded. The semi-structured interview guide was broad and asked standard questions about the participants’ background; how participants started work at the LTC facility; how participants dealt with stress, either in their job or family life; and how participants felt after a day’s work. In addition, various probes and prompts were also utilized after responses to these questions. For example, participants were asked to indicate how they cope, how they unwind, and/or to elaborate and tell the researcher more about the topic of interest after they discussed their response.

The goal of conducting observations and interviews was to gather rich data about the participants and their routines. For the quantitative component, an exploratory, paper-based pilot survey was distributed as well as a separate demographic questionnaire following each interview. The inclusion/exclusion criteria were that individuals must work within the health care sector in ancillary, support, or direct services, the worker’s roles must be directly related to the residential LTC facility. In all, 92 respondents filled and returned the survey, i.e., a response rate of 52% was achieved. One survey was excluded because the worker was not responsible for work that was related to any aspect of the LTC home. The purpose of the survey was to examine descriptive data such as smoking and alcohol consumption and the effects of sex or birth/immigration status. The survey asked participants whether they smoked cigarettes daily, occasionally, or not at all. The rationale was to assess whether or not tobacco use was light or heavy. Light or intermittent smoking was interpreted from the response “occasionally”, and heavy smoking was interpreted from the response “daily”. One person did not respond and was, therefore, excluded from the analysis. Mann–Whitney U tests were also conducted to test whether the distribution of being a cigarette smoker and the distribution of alcohol consumption was the same across categories of sex, immigrant status, and VM status. The survey also asked about alcohol consumption. Respondents were instructed to choose how often they drank alcoholic beverages over the past 12 months. There were seven choices to choose from, including an option to choose “not at all”. The rationale was to assess whether or not alcohol consumption was light/mild, moderate, or heavy. Light/mild alcohol consumption was interpreted from the responses “less than once a month”, “once a month”, “2 to 3 times a month”, while moderate consumption was interpreted from “once a week or 2 to 3 times a week”, whereas or heavy-alcohol consumption was interpreted from responses “4 to 6 times a week” or “every day”. One person did not respond to the survey and was excluded from the analysis. For the qualitative component, multiple units of analysis were organized by worker characteristics such as sex, job titles or roles, VM status, and employment type.

The term VM was used instead of non-White for several reasons. Firstly, the use of dichotomous terms such as White/non-White has been criticized in the research literature [28]. Secondly, there is often complexity with the use of such terminology. For example, sometimes South Asians self-identify as “White” based on skin color, which reflects cultural norms and values and contradicts/conflates what many researchers refer to as ethnically/racially “White.” Finally, there are issues of colorism that exist within different VM groups, such as Native American, Asian-American, and Latin-American communities (ibid). Accordingly, rather than utilizing White/non-White terminology extensively, in this study, VM/racialized, and non-VM/non-racialized are used, which are also utilized in Canadian official documents and arm’s-length agencies [29]. Racialized status refers to VMs, i.e., persons of color or non-White individuals who experience the process of racialization by which “societies construct races as real, different and unequal in ways that matter to economic, political and social life” [30].

Although qualitative and quantitative data collection was carried out concurrently, there was “separate data analysis” [31]. For the qualitative data analysis, fieldnotes and interview transcripts were analyzed with thematic analysis for the study using a selective coding system with the aid of NVivo computer software program to organize and sort information. Coding of data involved identifying themes and subcategories that were addressed in the interviews that were a part of the research questions. Codes were developed in consultation with other researchers, and some of these codes were also developed and used in previous LTC projects. The codes and the meaning of the themes were revised and amended as necessary, because coding is an iterative process. The coding list was compiled with the theoretical frameworks in mind, e.g., feminist political economy is reflected with codes such as gender, gender stereotyping, gendered work, and gender prejudice. The list of codes also included the following: immigration, immigrant, migrant, race, racialization, racism, prejudice, discrimination, support, and so forth. Quantitative statistical data analysis of the demographic questionnaire and survey occurred with the assistance of Excel and quantitative statistical software program (SPSS; Chicago, IL). Survey data were reviewed and checked by scholars from York University in consultation with the Statistical Consulting Service (SCS).

## 3. Results

Worker demographic data from the interviews and survey responses are indicated in Table 1 and Table 2. Typically, most LTC workers did not drink or smoke, though some did participate in these behaviors (Table 2).

### 3.1. Smoking and Alcohol Consumption

The survey results indicated that 92.2% (*n* = 83/90) of participants were non-smokers; however, 5.6% (*n* = 5/90) indicated “daily” smoking, and 2.2% (*n* = 2/90) indicated smoking “occasionally” (Table 3).

The survey results also indicated that 56.7% (*n* = 51/90) of participants reported not consuming alcohol at all (Table 4). The most frequent consumption pattern was 2 to 3 times a month (14.4%, *n* = 13/90), followed by less than once a month (12.2%, *n* = 11/90), which were light/mild patterns of consumption. No one reported consuming alcohol 4 to 6 times a week; however, one person reported consumption every day, which was interpreted as heavy consumption.

**Figure 2:**
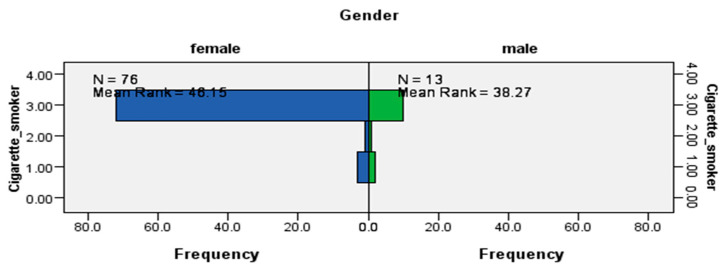
Independent-samples Mann–Whitney U test of the distribution of cigarette smoking across sex.

**Figure 3:**
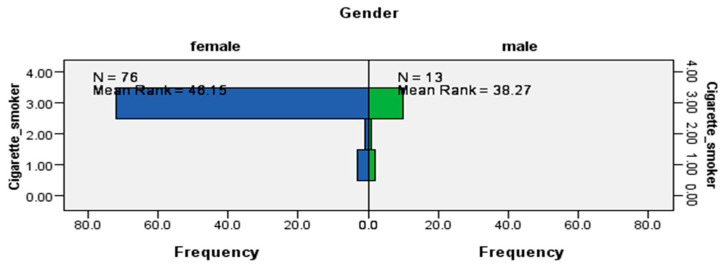
Independent-samples Mann–Whitney U test of the distribution of cigarette smoking across immigrant status.

The results from the Mann–Whitney U tests indicated that while the distribution of being a cigarette smoker was the same across visible minority status (data not shown), it was not the same for sex (U = 406.5, *p* < 0.0029) (Figure 2) and immigrant status (U = 732.5, *p* < 0.006) (Figure 3), which were statistically significant results. The data suggested that cigarette smokers from this LTC site were likely to be women and immigrants.

The survey responses pertaining to alcohol consumption are listed in Table 5 and Table 6. Overall, 56.7% (51/90) of the respondents reported not consuming alcohol at all, of which 90.2% (46/90) were female, and the same proportions were racialized (Table 5 and Table 6). The next most popular response was consuming alcohol 2–3 times a month (14.4%, 13/90), which was interpreted as light/mild consumption. 84.6% (11/13) of these latter respondents were female, and 76.9% (10/13) were racialized (Table 5 and Table 6). Only one female racialized respondent consumed alcohol every day, i.e., heavy consumption (Table 5 and Table 6).

**Figure 4:**
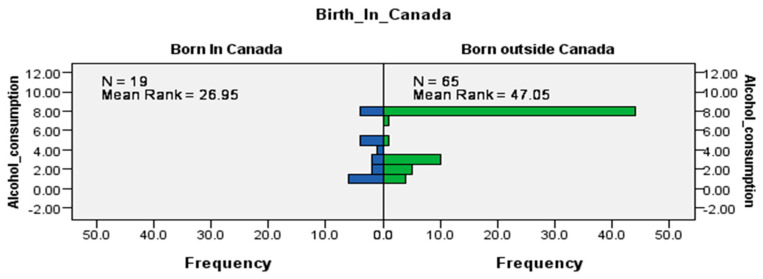
Independent-samples Mann–Whitney U test of the distribution of alcohol consumption across immigrant status.

The results of the Mann–Whitney U tests indicated that while the distribution of consuming alcohol was the same across sex and racial status (data not shown), it was not the same for Canadian-born workers and immigrants (U = 913, *p* < 0.001) (Figure 4), which were statistically significant results. Like cigarette smoking, alcohol consumption by workers at this LTC site was likely to occur among immigrants. These trends may be reflective of cultural differences between Canadian-born workers and immigrants or a combination of other factors, e.g., life experiences, the process of immigration, social support, and coping strategies that could play a role in alcohol consumption.

The qualitative data from observations did not provide significant findings with respect to smoking, alcohol consumption, stress-management techniques, or coping and defense mechanisms. However, interview data indicated that workers were exposed to high-stress environments and often employed a variety of coping mechanisms in order to minimize its effects. Interestingly, few participants disclosed smoking and alcohol consumption behaviors as a coping mechanism during face-to-face interviews. One possible reason for this trend might be that these activities are carried out as leisure activities as opposed to coping strategies. Another possible explanation is that there may be stigma associated with smoking and alcohol consumption, which may be perceived as socially unacceptable practices to discuss in workplace settings. The latter is important, especially given that the study was conducted in a health care environment, which could evoke additional pressure or stigma related to the health-related harms of smoking and alcohol consumption. This may have diminished disclosure among some interview participants during face-to-face interviews.

### 3.2. Coping, Defense, and Stress Management Techniques

#### Individual Methods of Coping and Defense

The data suggest that various health-impacting practices that may be utilized as coping, defense mechanisms, or stress management techniques were widely practiced by employees in the LTC home. For smoking and alcohol consumption, only a few participants reported these activities during interviews, e.g., Participant 5 (Director, Male, F/T), Participant 26 (Support Staff, Male, F/T). Participant 37 (Support Staff, Female, F/T), indicated consumption of alcohol to cope with a sleep disorder rather than taking prescription medication whereas Participant 40 (Ancillary Worker, Female, P/T) indicated consumption of alcohol for work-related stress management.

One worker indicated that sometimes she used a dramatic method to regain her peace of mind:

A: “Sometimes when I get too stressed, I go in the washroom and scream. Well it relieves me.” (Participant 16, PSW, Female, VM, F/T).

Another worker took brief timeouts to practice mindful breathing when work became too stressful because she said she was becoming unhealthy and had chest pain:

A: “I got sick of the stress. I had a lot of chest pains and I’m like, ‘No. I can’t not [sic] have stress get to me.’ When I close that door at 2 o’clock, I just have to let it go. And if I see something bothering me, I’ll just have to breathe […] Take my just two-minute break. I’ll just have a two minute.” (Participant 21, Ancillary Worker, Female, Non-VM, F/T).

When a support staff worker was asked about coping with stress, they indicated the practice of mindful breathing:

A: “Well, I take deep breaths, breathing technique, you know, and then sometimes I need like fresh air or cold air on my face, you know. I don’t know, probably to brush it off, but and just to be quiet, you know, and eat.” (Participant 27, Support Staff Worker, Female, VM, F/T).

Mindfulness and meditation practices were sometimes ritualized:

A: “I love reading. And then, I have a little ceremony. When I know that I’m feeling stressed, I have a—you know those candles, like [laughs] are they stress free or something, stress free candle. I have music. And I just lie down, just let loose of your body. Lie down, don’t think of anything. It’s a [sic] very good, like, yeah, meditation.” (Participant 2, Allied Health Worker, Female, VM, F/T).

A nurse used solitary contemplation to cope with stress:

A: “I don’t really meditate but I really just like to be on my own sometimes and just sit quietly in a room and not think about anything. I guess that’s my way of meditating, or just lay on the bed and just look at the ceiling, and just relax for like 10 min. Sometimes I fall asleep, sometimes I don’t.” (Participant 7, Nurse, Female, VM, P/T).

While the above participants used meditation and mindfulness as strategies of resilience, others indicated alternative strategies. When asked about how one deals with stress, a manager indicated that she would have moments where she would just break down and cry. The participant also said she listened to music, prayed, and had the support of her family. She reflected on religion in order to achieve a sense of purpose and perspective when confronting difficulties:

A: “I think I cope. I try to cope. There’s moments when I just break down and cry. Crying for me is a good thing. […] I pray. Prayer for me is a big thing. When I feel I’m too stressed, I pray. Praying helps a lot. Pray and music. […] And family support. I mean my parents, very, very supportive. My husband, great support.”

Many interview participants reported that they used solitary recreation as a coping strategy and indicated that they engaged in five forms of solitary recreation, including: watching television, reading, listening to music, engaging in a hobby (e.g., participants engaged in photography, singing, or playing the piano), and traveling. When a nurse was asked about relaxation, she said the following:

A: “As soon as I get home I just have to eat, watch my movies or watch my TV series for a little while and I’ll be fine. I’m back to myself.” (Participant 30, Nurse, Female, VM, F/T).

A manager watched movies on the home television and watched YouTube videos to purge negative emotions:

“Sometimes I put a movie, like a drama movie that I want to cry or watch YouTube videos that I just break in tears. Always my kids watch me […] they just look at me, ‘Okay, she’s going to cry now.’ They don’t know the stress of my day. I like music. Sometimes I just pump it so loud in the car and it just give [sic] me this relief” (Participant 32, Manager, Female, VM, F/T).

An ancillary worker said: “for mental repression I watch TV” (Participant 42, Ancillary Worker, Male, VM, F/T). A PSW said she liked to listen to music and watch movies to de-stress:

I: “You said you listen to music. Is that cultural music or—”

A: “Yeah, yeah. I’m [cultural background]. You know, movie—we have the movies. I love to listen some music; same time, I have our religious song, too. That’s especially Friday.” (Participant 36, PSW, Female, VM, F/T).

A nurse said she took short vacations when work-related stress became intolerable:

A: “I go away when it gets too much, I’m off somewhere, I’m just like itching, I need to get away. When I’m stressed I start to be very irritable […] I get tired, that’s when I get tired because I’m just like I’m done, I need a break and when that happens I know I need to go take time off or take a trip, I just need to get away because I’m boiling.”

I: “You like to go particular places or do you go any —?”

A: “Niagara Falls or take a trip to the [overseas country] or just stay home, take a—for a couple days I just stay home, but I can tell when my stress level is not normal, it’s getting—I’m antsy and I’m bitchy and I’m—I don’t do as much of production as if I’m—no I don’t focus and I get mad at every little thing and I—they say and I said be because I’m just fed up—no because it’s bad, I just—I’m stressed.” (Participant 38, Nurse, Female, VM, F/T).

### 3.3. Drawing on Social Support from Family, Friends, and the Community

#### Group Dynamics in Coping and Defense

While participants used mindfulness, prayers, and solitary recreation as ways to cope, others indicated that they had very little time for those sorts of strategies, and instead drew upon social support from family, friends, and the community as a source of stress relief. For example, an allied health worker reported that time spent with her young son provided her stress relief:

A: “My stress reliever’s [sic] my son, you know. I make sure I spend time with him, because if I just, you know, if I went to the gym or go to the gym, you know the time is short that you’re in, you should spend it with your son. You just read when he’s, you know, he’s playing, just read something that you know, self-help.” (Participant 3, Allied Health, Female, VM, F/T).

A manager used conversations with friends and sisters as a coping strategy. She indicated that previously, her stress levels were so high, it affected her health and sleep patterns, to the point that she eventually resigned from her previous job. She revealed that she was personally against the use of medication because of their side effects:

A: “Talking to my friends, you know, sometimes relieves the pressure. Talking and chatting. I have other sisters in other parts, not in Toronto, but you know, sometimes they visit and that kind of lightens the load a little bit. […] 15 years ago, or thereabouts, I had a breakdown, actually. A stressful thing because I was trying to manage everything […] And so, it all got to me, you know? Eventually. And it affected me, and I think also the—you give your best and when sometimes there’s no recognition, I think that also kind of affects your stress levels, you know? Or value given. So, I knew I had to stop. Actually, I stopped working, I actually resigned from my [previous] job. […] And I decided that I had to take care of my health because I had like, I couldn’t go to sleep and you know, I had all of those things. So, I kind of—I don’t believe in medication. Cause I know there are side effects” (Participant 6, Manager, Female, VM, F/T).

For one allied health worker, conversations with friends was helpful and relieved stress:

A: “I also—talking just to like my closest friends, sometimes, have [sic] been the best help. They don’t even have to say anything—just listening sometimes, you just get it off. And then, you can forget about [it], right, once you say it.” (Participant 14, Allied Health Worker, Female, Non-VM, P/T).

A nurse indicated that she took leave for vacations and drew upon support by calling and speaking with friends and coworkers so that they did not feel isolated in their situations in which she would “vent” to upper management to cope with the stress. She also indicated that her coworkers socialized with each other:

A: “I go on vacation. I just call up someone and I just vent. Or I vent to these people every day. Every day I see them I say this and this, and we walk in with that. Just to make it seem like we’re not alone. When you feel you’re not alone, it feels better. […] And then you know we’ll buy food once in a while, like when it’s someone’s birthday I’ll get her flowers. Like we’ll do things for one another. I close the door, I’ll sit with my manager, we vent to each other. I listen to her venting and I realize mine is nothing compared to hers. So yeah, it’s just the feeling of not being alone.” (Participant 17, Nurse, Female, VM, F/T).

While the above nurse felt she could confide in management, an allied health worker indicated she drew on support from her colleague:

A: “There’s one in my department—we’re really close. We support each other all the time. We’re always backing each other up. And whenever there’s a problem with the management, too, we back each other up.” (Participant 14, Allied Health Worker, Female, Non-VM, P/T).

A trainee also spoke of “venting” to her boyfriend to alleviate stress because she did not have time for anything beyond this strategy:

A: “So if anything my boyfriend hears a lot about my stress and usually just venting is enough for me.” (Participant 18, Trainee, Female, VM, F/T).

Another worker had friends who worked in other LTC facilities, and they would meet and commiserate to de-stress. Other than this, the participant indicated that perhaps stress was a part of the way she worked and lived now, indicating the level of stress that was normalized in her experience:

A: “venting—a lot of my personal—like my friends outside of work all work in long-term care. So, probably 90% of them work in long-term care and we just chat. You know, everybody has a best friend, you call your friend, you chat, you complain about the day. That’s about it. You know, the odd time you get together and over dinner as you meet with friends and everybody has a chance to complain about their job or talk about the funny things that happened at the job, that’s it. Other than that really it is just sometimes the best distress [sic] is just to sleep it away. […] Sometimes maybe it’s just stress is routine which is really scary but when you’re used to being in a stressful job I don’t think you know any different. So, I may not have a tool, I think it’s just the way I live my life now.” (Participant 22, Support Staff Worker, Female, Non-VM, F/T).

A different support staff worker said he would read a lot and talked with a close friend to “unwind”:

A: “I just—I read a lot. […] My roommate is a smart guy too, so we can have discussions about the world, or politics or stuff like that. […] That’s—I guess that’s how I unwind. […] And then there’s some guys that we hang out with on the weekends. Other than that, see the guys from back home every now and again.” (Participant 26, Support Staff Worker, Male, Non-VM, F/T).

## 4. Discussion

Previous research has suggested that in Canada, immigrants do not report elevated smoking intensity and alcohol consumption as coping strategies in response to work-related stress [19]. The findings presented throughout this study challenge previous work, in particular the patterns of smoking and alcohol consumption while revealing some new information about LTC workers’ health-impacting behaviors. For example, the data from this study suggests that while a majority of respondents were non-smokers and did not consume any alcohol, those few who did smoke were born outside of Canada and were women, and those who reported alcohol consumption were often immigrants, which challenges previous work that showed the opposite, i.e., that immigrants did not utilize these health-impacting behaviors to a significant degree. This finding about immigrants’ utilization of smoking/alcohol consumption may reflect complex interactions involving work in high-stress environments combined with cultural differences of particular immigrants and other factors (e.g., life experiences of women) that could play a role in these behaviors and may be a starting point of investigation for future studies.

Canadian studies of immigrants, racialized workers, and women suggest that these groups are often predisposed to high stress, chronic illness [32,33,34], and difficulties in managing domestic duties [35] due to work precarization [36], which adversely affects their health and well-being [33]. In addition, research has shown that in the care-work sector, many of these employees experience work hierarchies and strict divisions of labor [37], while utilizing various mechanisms to regulate emotional health [38]. This study confirms previous research about stress in LTC and reveals new information about stress management techniques, which may help to achieve or maintain workers’ social, mental, and physical well-being. Several strategies were identified including mindfulness, meditation, solitary recreation, as well as drawing on social support, and other personal health practices or coping mechanisms. Many participants indicated that they routinely engaged in mindful breathing techniques and meditation or solitary recreation as a source of stress relief. Others indicated that they simply did not have the time for these things and relied upon social support from family or friends. These findings shed light on under-researched areas of how social support and other adaptation processes are used by workers to engage with coping, resistance, and resilience. Understanding the particular cultural practices, resistance/resilience strategies, and how agency is expressed also points to possible directions in shifting the current framing of social determinants of health (SDoH) discourses that otherwise may not be reflective of ethnic, racial, or cultural groups.

Social support is one of the key SDoH [39]. The availability of social support as a key stress-adaptation process, and for coping and defense, may be crucial for workers so that they can access and exercise their preferred health/wellness practices, and how care workers realize their health and well-being, which may be especially important under significantly stressful conditions such as the current COVID-19 pandemic.

This study is not without its strengths and/or limitations. One of the limitations of this study was that it was conducted with a single-case (i.e., single site) investigation. Although such an approach means that the findings may not always be representative of and generalizable to provincial and national data, a strength of focusing on one site means that certain issues can be investigated in a deeper way, with a closer look at context. One of the strengths of this study is from its methodological approach. For example, mixed-methods research design is often rated for higher quality than other methods [40].

Finally, there are several policy implications of this study. Firstly, given that health care workers were found to exhibit various coping and defense mechanisms, it may be useful to conduct further studies to assess their success so that there are opportunities for vulnerable workers who can benefit by learning these strategies to utilize them. Furthermore, sharing this information may be beneficial as a part of the organizational-level recruitment and training process. Another policy option is to minimize stress at its root cause rather than treating stress. For example, workers may be experiencing stress because of the underlying issues of food insecurity, which has been previously reported in the care work literature [38]. Finally, income, employment, and working conditions are SDoH [39]. As one worker highlighted in this study, there is a need to recognize the value of care workers and to have mechanisms in place to reward and motivate them. Indeed this is an important point for organizations in which it is recommended that there are adequate provisions for intrinsic rewards such as interest, challenge, and personal satisfaction, and also extrinsic motivators (monetary/wages, salaries, tangible rewards) [41].

## 5. Conclusions

This work contributes to critical, interdisciplinary health scholarship by contextualizing social and behavioral health practices and coping mechanisms of workers in high-stress environments. The findings fill knowledge gaps in the health literature, while also suggesting typical health and lifestyle behaviors. For instance, workers reported smoking and alcohol consumption. However, these were rarely communicated in face-to-face interviews, possibly due to either stigma or because such consumption was not a coping mechanism but rather was for leisure or pleasure.

This study reveals the tension and high levels of stress found in LTC workers in the region of study as well as coping strategies that could promote health and wellness. Given that chronic stress and illness are strongly connected to each other, it would be beneficial to explore further follow-up studies in order to assess the effectiveness of chronic stress management. Another opportunity for future studies would be to further explore culturally unique practices for health and well-being. Furthermore, given that this study was small in scope, future research might include a survey with a wider scope and scale that includes other provinces and jurisdictions across Canada, and also seek to understand what types of assistance care workers may need, both within and outside of the home.

The evidence from this study demonstrates several important points. Firstly, it demonstrates that workers in the site of study often rely upon particular resources for support, such as for solitary recreation, in order to deal with the hazards stemming from their stressful, labor-intensive positions. The analysis also indicates that workers draw upon support from a variety of sources, including their co-workers.

While work stress and workloads in the LTC sector are known to be overwhelming, strategies to address these issues are often limited to behavioral modification, such as diet and physical activity interventions, rather than including a holistic approach, which considers income, employment, education (i.e., socioeconomic status), social support, and other SDoH. In order to manage care and care work (in this case, the care work that occurs in the LTC facility), it might be helpful to utilize particular initiatives that address and include SDoH, such as Total Worker Health©. Total Worker Health© initiatives aim to improve the health and well-being, of workers [42,43,44,45], through strategies that involve the home, family, and community of the workers. Given the diversity of care workers in the region of this study, such approaches would also need to be culturally appropriate, and adequate supports must be provided to the workers. This means that not only do services and provisions need to exist, but they also need to be available, affordable, and accessible to the workers who require them. When such services and support systems are made available to workers, they can perform the work better, safely, with less of a personal toll on their health and well-being, and with better outcomes for the recipients of care.

## Figures and Tables

**Table 1:** Interview participants’ characteristics (*n* = 42).

Characteristic		Frequency	%
Sex	Female	35	83.3%
	Male	7	16.7%
Employment Type	Full Time	32	76.2%
	Part Time	10	23.8%
Race/VM Status	Non-VM, non-racialized, White	12	28.6%
	VM, racialized	30	71.4%
Job Title/Role	Trainee	3	7.1%
	Allied Health	7	16.7%
	Nurse	9	21.4%
	Manager	4	9.5%
	Support Staff	6	14.3%
	Ancillary	6	14.3%
	Personal Support Worker (PSW)	7	16.7%

VM: visual minority.

**Table 2:** Descriptive statistics of the survey sample (*n* = 91).

Characteristic		Frequency	%
Sex	Female	76	83.5%
	Male	14	15.4%
	No response/omitted	1	1.1%
Birth/Immigration Status	Born in Canada	19	20.9%
	Born outside Canada, i.e., Immigrant	66	72.5%
	No responses/omitted	6	6.6%
Race/VM Status	Non-VM, non-racialized	11	12.1%
	VM, racialized	78	85.7%
	No response/omitted	2	2.2%
Smoking Status	Smoker	7	7.7%
	Non-smoker	83	91.2%
	No response/omitted	1	1.1%
Alcohol Consumption	Yes	39	43.40%
	No	51	56.7%
	No response/omitted	1	1.1%

**Table 3:** Smoking status.

Smoking Status	Frequency	%
Daily	5	5.6%
Occasionally	2	2.2%
Not at all, i.e., non-Smoker	83	92.2%
Total	90	100.0%

**Table 4:** Frequency of alcohol consumption.

Alcohol Consumption	Frequency	%
Less than once a month	11	12.2%
Once a month	7	7.8%
2 to 3 times a month	13	14.4%
Once a week	1	1.1%
2 to 3 times a week	6	6.7%
4 to 6 times a week	0	0.0%
Every day	1	1.1%
Not at all	51	56.7%
Total	90	100.0%

**Table 5:** Alcohol consumption by sex.

Alcohol Consumption			Sex			
	*n* = 90		*n* = 76		*n* = 13	
Response	Frequency	%	Female	%	Male	%
Less than once a month	11	12.2%	9	81.8%	2	18.2%
Once a month	7	7.8%	5	71.4%	1	14.3%
2 to 3 times a month	13	14.4%	11	84.6%	2	15.4%
Once a week	1	1.1%	0	0%	1	100%
2 to 3 times a week	6	6.7%	4	66.7%	2	33.3%
4 to 6 times a week	0	0%	0	0%	0	0%
Every day	1	1.1%	1	100%	0	0%
Not at all	51	56.7%	46	90.2%	5	9.8%
Total	90	100%	76	84.4%	13	14.4%

**Table 6:** Alcohol consumption by VM status.

Alcohol Consumption			VM Status			
	*n* = 90		*n* = 77		*n* = 11	
Response	Frequency	%	VM	%	Non-VM	%
Less than once a month	11	12.2%	9	81.8%	1	9.1%
Once a month	7	7.8%	7	100%	0	0%
2 to 3 times a month	13	14.4%	10	76.9%	3	23.1%
Once a week	1	1.1%	0		1	100%
2 to 3 times a week	6	6.7%	4	66.7%	2	33.3%
4 to 6 times a week	0	0%	0	0%	0	0%
Every day	1	1.1%	1	100%	0	0%
Not at all	51	56.7%	46	90.2%	4	7.8%
Total	90	100%	77	85.6%	11	12.2%
